# Effects of a Multimodal Lifestyle Intervention on Cardiometabolic Markers in People with Progressive Multiple Sclerosis: A Secondary Analysis of a Pilot Study

**DOI:** 10.3390/nu17071163

**Published:** 2025-03-27

**Authors:** Arturo S. Martinez, Alyanne J. Bastian, Farnoosh Shemirani, Tyler J. Titcomb, Babita Bisht, Warren G. Darling, Murali Ramanathan, Mujeeb Shittu, Christine M. Gill, Linda G. Snetselaar, Terry L. Wahls

**Affiliations:** 1 Department of Internal Medicine, University of Iowa, 200 Hawkins Dr, Iowa City, IA 52242, USA; arturo-martinez@uiowa.edu (A.S.M.); alyanne-bastian@uiowa.edu (A.J.B.); farnoosh-shemirani@uiowa.edu (F.S.); tyler-titcomb@uiowa.edu (T.J.T.); babita-bisht@uiowa.edu (B.B.); 2 Department of Epidemiology, University of Iowa, Iowa City, IA 52242, USA; linda-snetselaar@uiowa.edu; 3 Department of Health and Human Physiology, University of Iowa, Iowa City, IA 52242, USA; warren-darling@uiowa.edu; 4 Department of Pharmaceutical Sciences, State University of New York, Buffalo, NY 14214, USA; murali@buffalo.edu; 5 Department of Neurology, State University of New York, Buffalo, NY 14203, USA; 6 Department of Biotechnical and Clinical Laboratory Sciences, University at Buffalo, Getzville, NY 14214, USA; 7 Department of Neurology, University of Iowa Hospital and Clinics, Iowa City, IA 52242, USA; cmgill@uiowa.edu

**Keywords:** diet, apolipoprotein, comorbidity, multiple sclerosis, cardiometabolic markers

## Abstract

**Background**: Cardiometabolic comorbidities are common in multiple sclerosis (MS), and lifestyle interventions are effective in managing these conditions in the general population, though evidence in the MS patient population is limited. **Objective**: To evaluate the effect of a multimodal lifestyle intervention on serum apolipoproteins (Apo), creatine kinase (CK), glucose, and insulin in people with progressive MS (PwPMS). **Methods**: This study included *n* = 19 PwPMS who participated in a 12-month multimodal lifestyle intervention (including a modified Paleolithic diet, exercise, neuromuscular electrical stimulation, supplements, and stress reduction). Lipid profile (ApoA1, B, and E), CK, glucose, and insulin were obtained at baseline and after 12 months under fasting conditions. **Results**: At 12 months, there was a marginally significant decrease in ApoB (mean change: −7.17 mg/dL; 95% CI: −14.4, 0.12; *p* = 0.06), while no significant changes were observed for ApoA1 (mean change: −1.28 mg/dL; 95% CI: 12.33, 9.76; *p* = 0.80), ApoE (mean change: +0.12 mg/dL; 95% CI: −0.27, 0.52; *p* = 0.51), CK (mean change: +13.19 U/L; 95% CI: −32.72, 59.11; *p* = 0.55), Homeostasis Model Assessment of Insulin Resistance (HOMA-IR) (mean change: −0.44; 95% CI: −1.11, 0.22; *p* = 0.17), and HOMA-β (mean change: +45.62; 95% CI: −95.6, 186.9; *p* = 0.50). A positive association was observed between changes in HOMA-IR and fatigue changes at 12 months (β = 0.81, *p* = 0.02), suggesting that an increase in HOMA-IR was linked to increased fatigue, which was no longer significant following the exclusion of outliers (β = 0.71, *p* = 0.16). **Conclusions**: A multimodal lifestyle intervention did not negatively impact glycemic and lipid profiles. While improvements were observed in serum biomarkers, these changes were not statistically significant, highlighting the need for stronger evidence from larger, controlled studies to confirm the cardiometabolic health benefits in PwPMS.

## 1. Introduction

Multiple sclerosis (MS) is a chronic, immune-mediated neuroinflammatory disease with increasing prevalence both nationally and globally [1]. People with progressive MS (PwPMS) are characterized by pronounced neurodegeneration, brain and spinal cord atrophy, and steadily worsening disability [2]. The quality of life (QoL) of PwPMS is significantly diminished as disability advances [3,4]. In addition to MS-specific symptoms, comorbidities, including cardiometabolic conditions, are highly prevalent throughout the disease course [5] and are linked to greater severity of MS [6,7,8], brain volume loss [9,10], and death [11,12]. Emerging evidence suggests that adopting healthy lifestyle behaviors, including dietary modifications, regular physical activity, and mindfulness practices, either separately or in combination, is desirable among people with MS [13,14]. Several surveys have reported that over half of individuals with MS report implementing dietary modification [15,16,17,18]. Additionally, health behavior changes have shown promise in optimizing disease outcomes [19,20,21] and managing comorbid conditions [22,23].

In MS, comorbidities refer to the presence of additional health conditions that coexist alongside MS and require medical care [24]. Cardiometabolic comorbidities are common among PwMS, and their management and prevention are suggested to be a pressing clinical concern [25]. For instance, impairments in lipid and glucose profiles can lead to poor MS disease prognosis [26], specifically with increased total cholesterol (TC), low-density lipoprotein (LDL), triglyceride (TG), and insulin levels being associated with increased MS disability progression [27]. Notably, cardiometabolic comorbidities are often considered modifiable conditions, which could be prevented or improved by lifestyle modifications [28]. While the current evidence preliminarily indicates the promising impact of individual lifestyle factors, including adopting a healthy diet [29,30,31,32,33], exercise training [34,35], and mindfulness therapies [36,37], on clinical and patient-reported outcomes, only sparse evidence exists for how multimodal lifestyle modification interventions impact MS symptoms, as well as cardiometabolic risk factors among PwMS [38,39].

Evidence indicates a link between TC and other lipid biomarkers and disease progression in MS [40,41]. Biomarkers related to the cholesterol pathway, including HDL-C and LDL-C levels, apolipoproteins (ApoA1, ApoAII, ApoB, and ApoE), and other cholesterol metabolites, have been linked to the disruption of the blood–brain barrier [42] and neurodegeneration in MS [9]. In this regard, apolipoproteins, proteins that mediate carriage of cholesterol and other lipids in the serum, are important factors in cholesterol homeostasis and have been shown to be associated with neuroaxonal injury, contributing to MS progression [43,44]. For instance, ApoE is the dominant apolipoprotein in the brain, and its role in MS has been extensively studied [45,46]. Additionally, ApoA1 is involved in HDL biosynthesis and transport, and has a neuroprotective effect on the immune and central nervous systems [47], while ApoB, the major component of LDL, is independently associated with higher Expanded Disability Status Scale (EDSS) scores [26].

Several previous studies have demonstrated the beneficial impact of lifestyle interventions, including diet and exercise, on lipid and glycemic profile biomarkers among people with cardiometabolic conditions [48,49,50]. However, limited research has investigated these effects within the MS community [31,51,52,53]. Notably, our prior work using the data from the same trial [39] showed that this 12-month multimodal intervention including diet, exercise, neuromuscular electrical stimulation, and stress reduction significantly decreased LDL and TG levels as well as increasing HDL levels, which were associated with improvements in fatigue [52]. Additionally, evidence suggests that disruptions in insulin signaling could contribute to the development of neurodegenerative disorders like MS. A recent meta-analysis found that individuals with MS have significantly higher insulin resistance (IR) compared to healthy people, which was more pronounced among individuals with progressive MS, likely due to increased oxidative stress [54]. Building on previous findings regarding the contribution of improvements in lipid markers due to a change in diet and reductions in fatigue [31,52,53,54,55] and the established link between IR and MS [56,57,58], this secondary analysis seeks to further explore the impact of diet-based lifestyle interventions on cardiometabolic health and explore their potential associations with fatigue. We hypothesize that a multimodal lifestyle intervention combining diet, physical activity, stress management, and neuromuscular electrical stimulation will lead to improvements in glycemic and lipid biomarkers. The results of this study could contribute to new practices by promoting the integration of lifestyle modification interventions into MS clinical care. By linking improvements in lipid and glycemic profiles with better clinical outcomes, this study may inform personalized, interdisciplinary strategies for PwPMS.

## 2. Materials and Methods

### 2.1. Study Population and Design

The study data of this secondary analysis were obtained from a previously conducted pilot trial studying the impact of a multimodal lifestyle intervention on fatigue over a 12-month period in a sample of 20 PwPMS [39]. The intervention included a modified Paleolithic diet, neuromuscular electrical stimulation, exercise, supplements, and stress reduction. In the present secondary analysis among (*n* = 19) participants, apolipoprotein and glycemic profile samples were analyzed at baseline and 12-month follow-up.

### 2.2. Inclusion and Exclusion Criteria

The inclusion criteria for the study were as follows: (1) a diagnosis of progressive MS confirmed by a neurologist specializing in MS, (2) the presence of gait impairment while still being able to walk 25 feet independently or with an assistive device, (3) age 18–65 years old, and an adult companion willing to assist with home exercises and neuromuscular electrical stimulation. The exclusion criteria for the study included the following: (1) a change in the MS diagnosis within the previous 3 months, (2) an active cancer diagnosis (excluding non-melanoma skin cancer), (3) significant cognitive dysfunction, seizure disorders, psychotic disorders, abnormal hepatic or renal functions, (4) abnormal heart rhythm or heart block, (5) unstable heart disease, antiplatelet or anticoagulant medication, (6) lung disease, diabetes requiring changes in medication in the prior three months, (7) an implanted electronic device, and vitamin D levels exceeding 150 ng/mL (or levels above 100 ng/mL accompanied by elevated blood calcium levels over 10.2 mg/dL). Notably, fatigue status was not included in the eligibility criteria for this study [52].

### 2.3. Study Protocol

The University of Iowa Human Subjects Institutional Review Board provided ethical approval (IRB#201611800) of the study and its protocol on 25 May 2010. All participants provided written informed consent. This study is registered under ClinicalTrials.gov identifier NCT01381354 [39]. Before the intervention began, participants completed a two-week run-in phase focused on educating them about the study’s dietary protocol. During the run-in period, participants were asked to follow the study diet, perform exercise stretches from a stretching exercise program designed for each of them, and keep records of their food intake and physical activity. A trial electrical stimulation session was performed during the second visit. Those who were able to tolerate the electrical stimulation and adhered to the diet for seven consecutive days during the run-in phase were eligible for inclusion in the 12-month study [39].

### 2.4. Study Diet

The diet component was the foundation of the multimodal intervention, outlining specific foods to prioritize (recommended), avoid (excluded), and incorporate in moderation (encouraged). Participants were guided to consume three daily cup-equivalent servings of sulfur-rich vegetables, leafy greens, and vibrantly colored vegetables and fruits. Foods to avoid included gluten-containing grains, dairy, and eggs. The diet also promoted daily consumption of animal protein (at least 4 ounces), plant-based protein sources (at least 4 ounces), omega-3-rich oils (2 tablespoons), and non-dairy milk alternatives such as soy, almond, rice, or coconut milk. Additionally, participants were encouraged to include nutritional yeast, kelp, and algae supplements like spirulina and chlorella. Intake of gluten-free grains and starchy foods was limited to two servings per week. Participants were advised to eat to fullness.

Weight loss or fasting behaviors were not the goal of this study. If a participant lost 10% of their body weight or more while part of the study, their primary care physician was notified, and the study team worked with the participant to increase higher-calorie foods in their daily intake [35]. Participants were advised to take dietary supplements believed to benefit MS and fatigue but were free to refuse or discontinue them at any time [39].

### 2.5. Exercise, Neuromuscular Electrical Stimulation, and Stress Reduction

Each participant was provided with a personalized home-based exercise program targeting leg and trunk muscles, which included both stretching and strengthening exercises. The majority of strengthening exercises were combined with neuromuscular electrical stimulation (NMES) to enhance muscle contraction and movement. Initially, participants performed 10–20 repetitions of each exercise within 10 min of electrical stimulation. As participants’ tolerance improved, the number of repetitions and duration of both exercises and electrical stimulation were gradually increased. Participants were instructed to engage in stretching and exercise–NMES at least five days a week. Detailed descriptions of the exercise types and electrical stimulation protocols have been provided in prior publications [52]. For stress reduction, participants were instructed to practice mantra-based meditation and self-massage their hands, feet, and face for a recommended duration of 20 min daily [39].

### 2.6. Biochemical Marker Assessment

Serum biochemistry analytes, including glucose and creatine kinase, were measured using diagnostic reagent kits, calibrators, and quality control materials from Sekisui Diagnostics (Burlington, MA, USA). Apolipoproteins (ApoA1, B, and E) and insulin were measured using diagnostic immunoturbidometric reagent kits, calibrators, and quality control materials from Kamiya Biomedical Co. (Seattle, WA, USA). All assays were adapted to the ABX Pentra 400 automated chemistry analyzer (Horiba Medical, Irvine, CA, USA). Additionally, Homeostasis Model Assessment of Insulin Resistance (HOMA-IR) was calculated as fasting glucose (mg/dL) multiplied by fasting insulin (μU/mL) divided by 405 [59], where a value of <2 is considered to be insulin-sensitive and a value ≥ 2 is considered to be insulin-resistant. HOMA-β, which assesses beta cell function from fasting glucose and insulin concentrations, was calculated as 360 × fasting insulin (µU/mL)/[fasting glucose (mg/dL) − 63]. All samples were collected in a fasting state in the morning before breakfast.

### 2.7. Statistical Analysis

A paired *t*-test was conducted to evaluate changes in creatine kinase (CK), apolipoprotein (ApoA1, ApoB, and ApoE) and glucose (glucose and insulin) profile variables between baseline and 12 months. The association of change in Fatigue Severity Score (FSS) with changes in cardiometabolic variables (ApoA1, ApoB, ApoE, glucose, insulin, and CK) from baseline to 12 months was assessed using general linear models, adjusting for baseline values. The change in FSS from baseline to 12 months served as the dependent variable, while the change in the selected cardiometabolic variable over the same period was treated as the independent variable. No corrections for multiple comparisons were applied, as this analysis is exploratory in nature, aiming to explore potential relationships for future investigations.

## 3. Results

### 3.1. Demographic Characteristics

**Figure 1:**
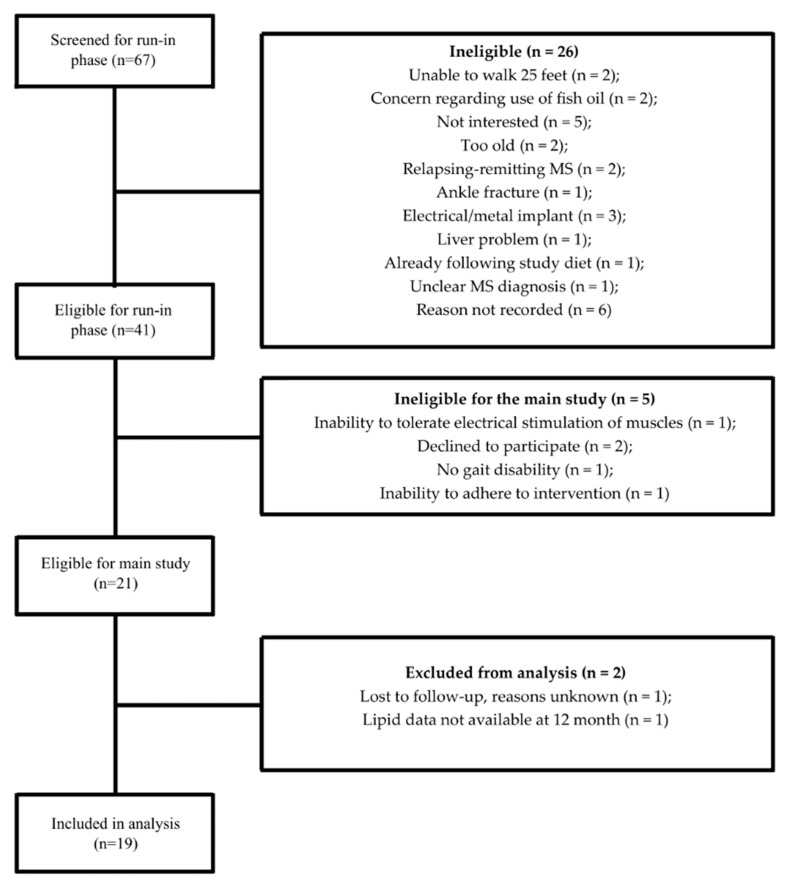
CONSORT diagram for the cardiometabolic marker sub-study.

The detailed information of the study participants has been explained in detail elsewhere [39]. Briefly, 19 progressive MS patients were included in the present secondary analysis. Figure 1 shows the CONSORT diagram for the present sub-study.

Table 1 displays the baseline clinical and demographic characteristics of the participants. As this was a cohort of PwPMS, the majority (*n* = 14, 73.7%) used a walking aid. Sixteen participants had a baseline FSS score ≥ 4.0. Nine of the nineteen subjects (47.4%) were taking approved disease-modifying therapies (DMTs). Treatment choices were determined by the patients’ attending neurologists, and no adjustments to their DMTs were introduced as part of the study.

### 3.2. Cardiometabolic Marker Change

**Figure 2:**
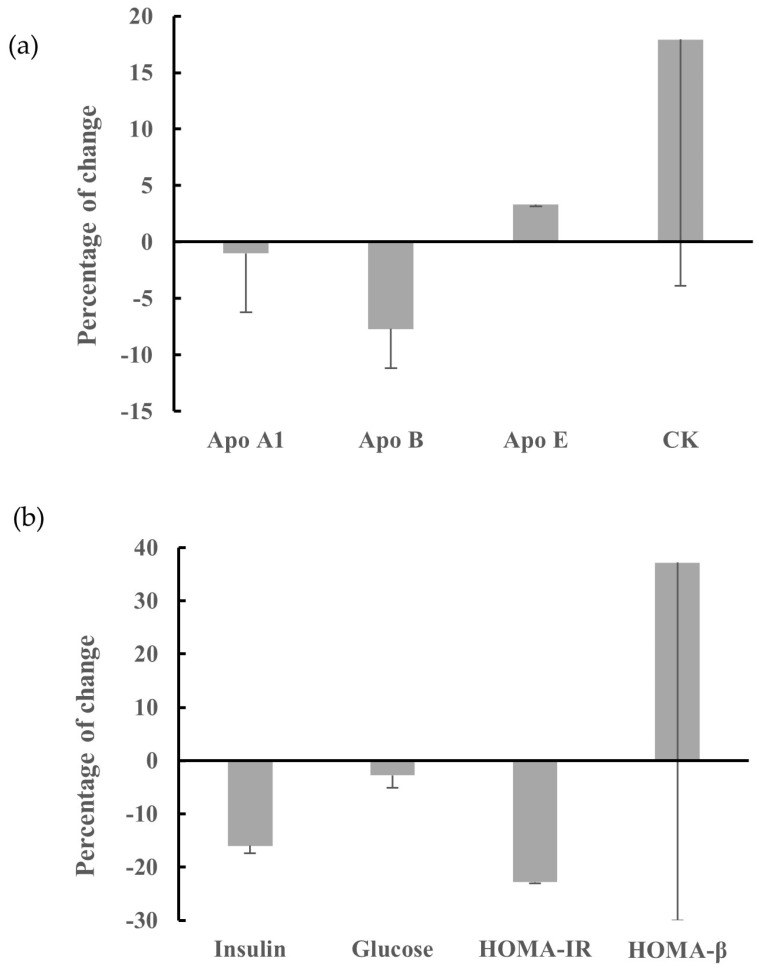
Percentage of change from baseline for serum cardiometabolic markers including: (**a**) ApoA1, B, E, and CK; and (**b**) insulin, glucose, HOMA-IR, and HOMA-β, following multimodal intervention among *n* = 19 participants.

As shown in Figure 2, ApoB showed a marginally significant decrease at 12 months compared to baseline (mean change: −7.17 mg/dL; 95% CI: −14.4 0.12; *p* = 0.06), while no changes were observed for ApoA1 (mean change: −1.28 mg/dL; 95% CI: −12.33, 9.76; *p* = 0.80), ApoE (mean change: +0.12 mg/dL; 95% CI: −0.27, 0.52; *p* = 0.51), CK (mean change: +13.19 U/L; 95% CI: −32.72, 59.11; *p* = 0.55), glucose (mean change: −2.51 mg/dL; 95% CI: −7.42, 2.39; *p* = 0.29), HOMA-IR (mean change: −0.44; 95% CI: −1.11, 0.22; *p* = 0.17), HOMA-β (mean change: +45.62; 95% CI: −95.6, 186.9; *p* = 0.50), or insulin (mean change: −1.34 μIU/mL; 95% CI: −4.30, 1.60; *p* = 0.34). We also performed analyses excluding subject IDs 5 and 18 as outliers. Subject ID 5, diagnosed with chronic lymphocytic leukemia (CLL) (a consequence of prior mitoxantrone use for SPMS), exhibited an unexpected sharp rise in serum insulin (Figure S1). Subject ID 18 had the highest disability level at baseline (EDSS = 8) and demonstrated a high rate of progressive decline after diagnosis [39]. However, the exclusion of these two subjects did not alter any of the findings. Additionally, mean changes from baseline at 6 months showed no significant differences in any biomarkers or their associations with fatigue (Tables S1 and S2).

Changes in HOMA-IR were significantly associated with changes in FSS at 12 months among *n* = 19 (including subject IDs 5 and 18), with β-coefficients (95% CI) of 0.81 (0.12, 1.5; *p* = 0.02; Table 2). However, after excluding subject IDs 5 and 18, the association was no longer significant (β = 0.71 (−0.33, 1.75; *p* = 0.16); Figure S2). No significant associations were observed between changes in apolipoproteins and CK and changes in FSS (Table 2).

## 4. Discussion

The findings of the current secondary analysis of a previous pilot study suggest that a multimodal lifestyle intervention including a modified Paleolithic diet, exercises, neuromuscular electrical stimulation, stress management techniques, and supplementation may lead to a modest reduction in ApoB levels. Furthermore, ApoE, CK, and HOMA-β demonstrated a non-significant upward trend, while HOMA-IR exhibited a downward trend. Additionally, a positive association was found between the change in insulin resistance and the change in fatigue, suggesting that improvements or worsening in insulin resistance may be correspondingly linked to improvements or worsening in fatigue; however, after excluding outliers from the analyses, the association between insulin resistance and fatigue was no longer statistically significant.

Comorbidities are increasingly prevalent in MS [5]. Growing evidence suggests that unhealthy lifestyle habits, including poor diet quality, stress, and physical inactivity, negatively affect disability in PwMS [60,61] and are associated with greater risk of comorbidities, such as cardiovascular diseases [62]. Previous studies have shown that PwMS who had one or more cardiovascular risk factors had an increased lesion burden and more advanced brain atrophy [63]. In addition to their clinical impact, the presence of comorbid conditions in individuals with MS has been associated with diagnostic delays and increased hospitalizations [64]. Adopting health behaviors, including a healthy diet and moderate exercise, have been shown to be associated with a decreased risk of comorbidities and increased likelihood of higher QoL [65].

Increasing research has focused on the impact of lifestyle modification interventions, whether implemented individually or in combination, on clinical and patient-reported outcomes, along with lipid and glucose profiles, yielding encouraging results [23,38,66]. Findings from the original pilot study, which included the population analyzed in this sub-study, showed that adopting a diet-based multimodal intervention over 12 months led to significant improvements in fatigue, QoL [39], mood, and cognition [67]. Additionally, reductions in fatigue were linked to elevation in HDL-C and changes in TC over the 12-month period [52]. In the present study, both insulin and glucose exhibited a decreasing trend at 12 months, though the changes were not statistically significant. Furthermore, a positive association was observed between insulin resistance (calculated as HOMA-IR, which incorporates both fasting insulin and glucose) and changes in fatigue (measured as FSS score). Specifically, for every one-unit increase in HOMA-IR (worsening insulin resistance), fatigue (FSS score) is predicted to increase by 0.81 units. This suggests that higher insulin resistance is associated with higher fatigue. A positive slope was observed both with all data included and after excluding two subjects, indicating a positive association between changes in insulin resistance and fatigue in both cases. However, after excluding these two subjects—who appeared to be the primary drivers of the relationship—the association was no longer significant. Similarly, when all participants were included, HOMA-β (β-cell function) increased from 123% to 168%, remaining within the normal range. However, after excluding IDs 5 and 18, HOMA-β showed a slight decrease (from 104% at baseline to 90% after the 12-month intervention).

The finding of improved insulin levels observed in the present study is consistent with prior research from the Wahls research team, which demonstrated that a modified Paleolithic elimination (Wahls) diet resulted in statistically and clinically significant reductions in insulin levels at 12 and 24 weeks compared to a low-saturated fat (Swank) diet group [53]. However, no association was found between changes in insulin and changes in fatigue status in that study. These findings also align with other studies indicating that a Paleolithic diet significantly lowers plasma insulin and enhances insulin sensitivity in obese participants without MS [68].

In the present study, the apolipoprotein profile showed non-significant improvements, a decrease in ApoB and an increase in ApoE, and no associations with changes in fatigue. Based on the significant improvements found in HDL-c and LDL-c in a previous secondary analysis of the same sub-population [52], we had expected to potentially observe similar significant improvements in their corresponding apolipoproteins. However, these results might be attributed to the fact that changes in apolipoprotein levels may take longer to manifest due to differences in regulatory mechanisms, slower turnover rates of apolipoproteins, and the distinct effects of diet on lipids compared to proteins. Consistent with our findings, a study examining the effects of the Mediterranean–DASH Intervention for Neurodegenerative Delay (MIND) diet, promoting the intake of healthy fats, particularly monounsaturated fats and omega-3s, on biochemical markers and clinical outcomes in PwMS also observed significant reductions in LDL-c and TG, along with improvements in clinical outcomes. However, similar to our study, no significant changes in ApoA1 and ApoB levels were observed [69]. Additionally, in another four-month pilot study analyzing the relationship between lipid profile, functional disability, and fat consumption in individuals with MS following intervention with epigallocatechin gallate and coconut oil, it was found that TG levels decreased in the intervention group compared to the control group, which was positively correlated with an improvement in functional disability, as assessed by the EDSS, and negatively correlated with HDL and ApoA1 levels [70].

In another study by Niesten et al., the cardiometabolic health effects of replacing sitting with light-intensity physical activity throughout the day were compared to a single bout of vigorous-intensity exercise in PwMS [71]. This randomized cross-over study found that both light-intensity physical activity and vigorous-intensity exercise led to significant improvements in insulin sensitivity, blood lipids, and inflammatory markers compared to prolonged sitting. Notably, there was no significant change in ApoA1 compared to the control, while similarly to our results, ApoB decreased following light-intensity physical activity compared to sitting [71].

Investigating changes in apolipoproteins in the context of MS holds great importance due to their association with disease outcomes and progression, as well as their potential role as an accurate biomarker for assessing cardiovascular disease [72,73]. A systematic review, examining the relationship between levels of cholesterol and markers of cholesterol turnover and MS disease outcomes, suggests that elevated levels of circulating LDL-C, TC, and particularly ApoB are associated with adverse clinical and MRI outcomes in MS [73]. Greater ApoB levels have been shown to be associated with an increased number of new or enlarging T2 lesions over a two-year period in clinically isolated syndrome [74]. Additionally, ApoB, the key structural protein of atherogenic lipoproteins, may serve as a reliable marker for assessing vascular injury. The findings of this study are significant, as they emphasize the role of lifestyle modifications in improving not only MS-specific outcomes but also a few markers of vascular comorbidities, underscoring the need for further investigation.

In the present study, a non-significant increase in serum CK levels was observed following multimodal intervention. However, evidence regarding the impact of lifestyle behavior changes on serum CK levels in the context of MS is limited. Previous research has shown that CK activity is significantly lower in individuals with MS compared to healthy controls [69,75]. Our findings align with a study investigating the biochemical effects of the MIND diet in people with MS, which also reported a non-significant increase in serum CK levels following a 12-week dietary intervention [69]. Reduced CK activity in MS may be attributed to decreased muscle activity, as physical activity often declines due to factors such as weakness, fatigue, medication, or disability related to the disease itself [75].

The present secondary analysis was limited by the pilot trial’s unblinded single-arm design, which involved a small sample size of 19 participants with progressive MS. It should be emphasized that all participants were instructed to take up to two tablespoons of omega-3 fats in addition to the study diet. The observed improvements in the lipid profile may be attributed to the omega-3 supplementation. Additionally, another limitation of this study is the difficulty in obtaining reliable measurements of CK. CK levels can exhibit significant individual variability due to factors such as physical activity, type of exercise, underlying muscle mass, and other physiological conditions. This variability can affect the interpretation of CK data and may obscure potential effects of the intervention. Future studies should consider strategies to account for this variability, such as standardizing physical activity levels, volitional and neuromuscular electrical-stimulation-augmented exercise duration and intensity, body composition assessments prior to measurements, or using additional biomarkers to corroborate CK findings.

Overall, this study suggests improvement in cardiometabolic markers following a 12-month intervention. While we acknowledge that the clinical impact of these findings is limited, there are several factors which could explain the lack of significant effects: (1) the small sample size of 19 participants may have limited the statistical power to detect significant changes; (2) the 12-month duration of the intervention may not have been sufficient to observe significant changes in some cardiometabolic markers, which may require longer intervention periods to show measurable effects; (3) the participants’ baseline health status, for example the presence or development of new comorbidities (e.g., CLL), may have influenced their response to the intervention; and (4) the sensitivity of the biomarkers used to assess cardiometabolic health may vary. Some markers may not be as responsive to lifestyle changes as others, and more sensitive or additional biomarkers could be considered in future research. Thus, while the data observed in our study are promising, the limited clinical impact underscores the need for future research with larger sample sizes, extended follow-up periods, inclusion of a control group, and a more comprehensive set of biomarkers to fully evaluate the potential benefits of multimodal lifestyle interventions in PwPMS.

## 5. Conclusions

In conclusion, the current study demonstrated a marginally significant reduction in ApoB levels. Additionally, ApoE, CK, and HOMA-β exhibited a non-significant favorable upward trend, while HOMA-IR showed a decreasing trend, suggesting a potentially beneficial response. Furthermore, a positive association was observed between changes in insulin resistance and changes in fatigue, highlighting the potential role of lifestyle interventions in improving glycemic control and lipid profiles in individuals with MS. Although we did not observe significant mean changes in cardiometabolic markers from baseline, it is worth noting that no significant negative changes were observed over the 12-month period either. Future controlled clinical trials are warranted to confirm and validate these findings.

## Figures and Tables

**Table 1:** Characteristics of study participants ^a^.

Characteristics	*n*	Mean ± SD or *n* (%)
Age, years	19	51.0 ± 6.5
Sex, % female	19	14 (73.7)
BMI, kg/m^2^	19	24.4 ± 3.0
Race	19	
Caucasian		18 (94.7)
Hispanic		1 (5.3)
Education	19	
High school		1 (5.3)
Some college		6 (31.6)
Four-year degree		4 (21.0)
College		8 (42.1)
Diagnosis	19	
SPMS		17 (89.5)
PPMS		2 (10.5)
Walking aid use	19	14 (73.7)
Disease duration, years	19	13.6 ± 7.5
Disability (EDSS)	19	6.2 ± 1.0
Fatigue (FSS)	19	5.5 ± 1.3
DMTs	19	9 (47.4)

^a^ Adapted with permission [52]. Abbreviations: BMI: Body Mass Index; DMTs: disease-modifying therapies; EDSS: Expanded Disability Status Scale; FSS: Fatigue Severity Scale; *n* = numbers; SD: standard deviation; SPMS: secondary progressive MS; PPMS: primary progressive MS.

**Table 2:** Association of 12-month changes in fatigue with cardiometabolic marker change among *n* = 19 participants with progressive MS following a multimodal intervention.

	FSS	
	β-Coefficient (95% CI)	*p*-Value
ApoA1	−0.00 (−0.03, 0.03)	0.99
ApoB	−0.02 (−0.06, 0.03)	0.43
ApoE	−0.57 (−1.42, 0.26)	0.16
HOMA-IR	0.81 (0.12, 1.5)	0.02
HOMA-β	0.002 (−0.000, 0.004)	0.06
CK	−0.00 (−0.01, 0.01)	0.53

Data analyses among *n* = 19. The β-coefficient is the degree of change in the outcome variable for every 1 unit of change in the independent variable. Abbreviations: Apo: apolipoprotein; CK: creatine kinase; FSS: Fatigue Severity Scale.

## Data Availability

All relevant data are within the manuscript, the tables, and figures.

## Supplementary Materials

The following supporting information can be downloaded at: https://www.mdpi.com/article/10.3390/nu17071163/s1. Figure S1: Serum insulin levels for individual participants (identified by ID) before and following 12-month multimodal intervention. Participant ID 5 was undergoing treatment for CLL. Figure S2: Scatter plot depicting the relationship between changes in fatigue and HOMA-IR among *n* = 19 (blue line and circles; β = 0.81, *p* = 0.02) and *n* = 17 (red dashed line and triangles; β = 0.71, *p* = 0.16), excluding subject IDs 5 and 18. Table S1: Mean changes from baseline for serum cardiometabolic markers following multimodal intervention at 6 months and 12 months. Table S2: Association of 6-month changes in fatigue with cardiometabolic markers change among individuals with progressive MS following a multimodal intervention.

## Abbreviations

The following abbreviations are used in this manuscript:

BMI	Body Mass Index
CIS	Clinically isolated syndrome
CK	Creatine kinase
CLL	Chronic lymphocytic leukemia
CNS	Central nervous system
CPB	Cholesterol pathway biomarkers
DMT	Disease-modifying treatment
EDSS	Expanded Disability Status Scale
EGCG	Epigallocatechin gallate
EX	Vigorous-intensity exercise
FSS	Fatigue Severity Scale
HDL	High-density lipoprotein
HOMA-IR	Homeostasis Model Assessment of Insulin Resistance
IR	Insulin resistance
LDL	Low-density lipoprotein
LIPA	Light-intensity physical activity
MIND	Mediterranean–DASH Intervention for Neurodegenerative Delay
MRI	Magnetic resonance imaging
NMES	Neuromuscular electrical stimulation
PP-MS	Primary progressive multiple sclerosis
PwMS	People with multiple sclerosis
SP-MS	Secondary progressive multiple sclerosis
TC	Total cholesterol
TG	Triglyceride
