# Supplementary material for: Effects of a Multimodal Lifestyle Intervention on Cardiometabolic Markers in People with Progressive Multiple Sclerosis: A Secondary Analysis of a Pilot Study
Source: Nutrients. 2025 Mar 27;17(7):1163. doi: 10.3390/nu17071163 (PMC11990591; doi:10.3390/nu17071163)
Supplement: Supplementary file 1 [file nutrients-17-01163-s001.zip › nutrients-3517892-supplementary.pdf]

Figure S1

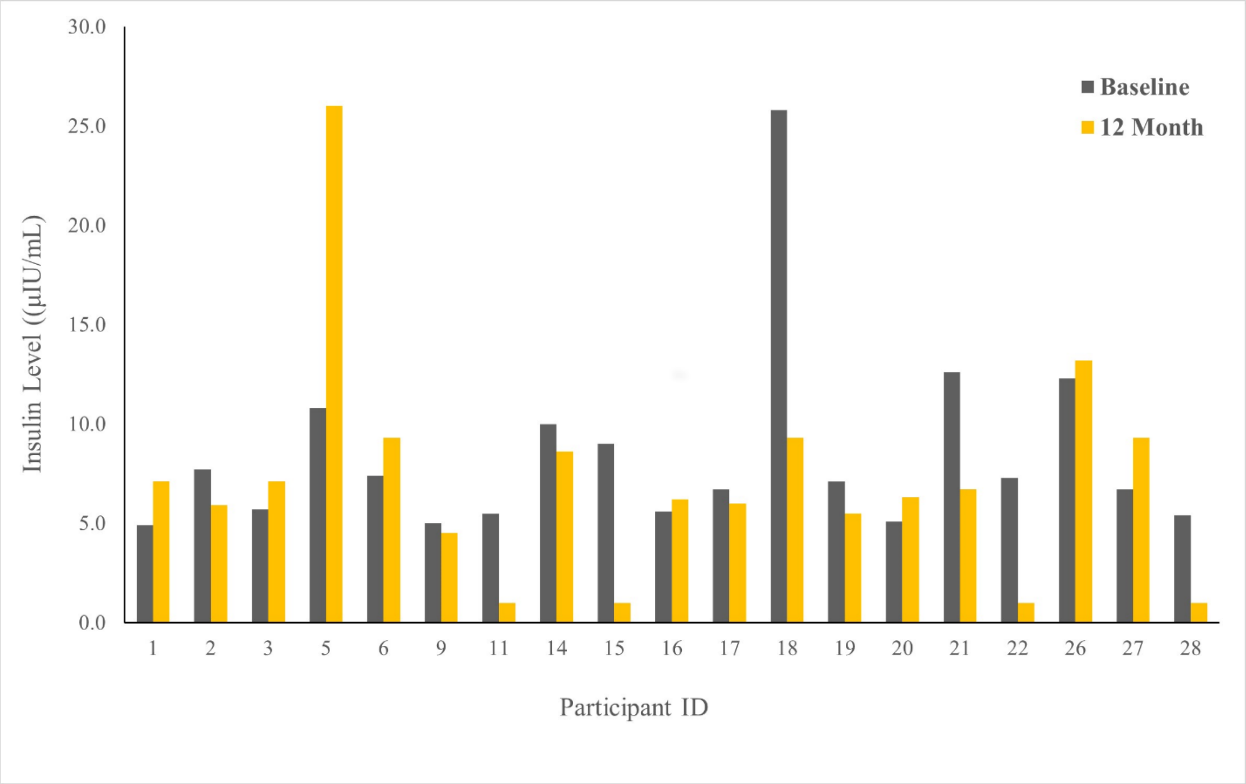

Figure S2

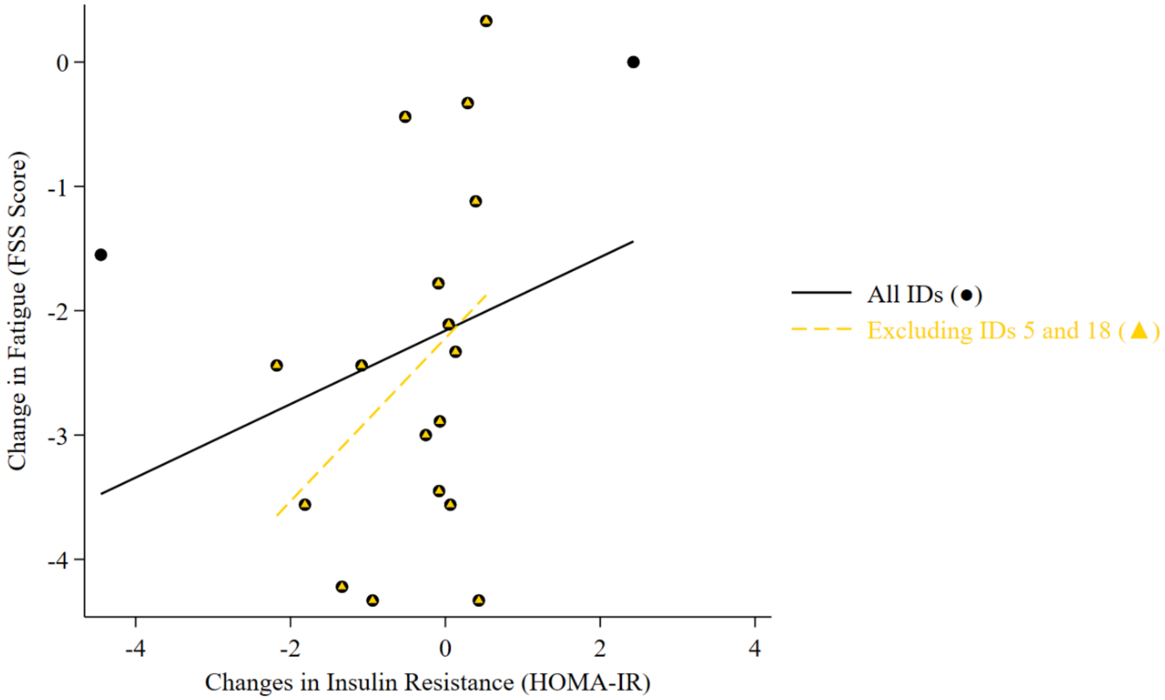

Table S1. Mean changes from baseline in serum cardiometabolic markers following multimodal intervention at 6-month and 12-month[1].

Biomarker		Mean change from baseline	95% CI	p-value[2]
**Glucose (mg/dL)**	6-Month	-0.35	-7.24, 6.54	0.10
	12-Month	-2.51	-7.42, 2.39	0.29
**Insulin (μIU/mL)**	6-Month	-0.23	-3.11, 2.64	0.90
	12-Month	-1.34	-4.30, 1.60	0.34
**HOMA-IR**	6-Month	-0.13	-0.82, 0.55	0.70
	12-Month	-0.44	-1.11, 0.22	0.17
**HOMA-β**	6-Month	-20.40	-121.64, 80.82	0.70
	12-Month	45.62	-95.6, 186.9	0.50
**CK (U/L)**	6-Month	-2.57	-22.1, 16.98	0.80
	12-Month	13.19	-32.72, 59.11	0.55
**Apo A1 (mg/dL)**	6-Month	-1.44	-10.49, 7.60	0.75
	12-Month	-1.28	12.33, 9.76	0.80
**Apo B (mg/dL)**	6-Month	-5.59	-11.63, 0.45	0.07
	12-Month	-7.17	-14.4, 0.12	0.06
**Apo E (mg/dL)**	6-Month	0.05	-0.32, 0.42	0.80
	12-Month	0.12	-0.27, 0.52	0.51

[1] Data from N=19 participants.

[2] Obtained from paired t-test.

Table S2. Association of 6-month changes in fatigue with cardiometabolic markers change among individuals with progressive MS following a multimodal intervention[1].

	**FSS**	
	**β-coefficient (95% CI)**	**p-value**
**ApoA1**	0.01 (-0.02, 0.04)	0.50
**ApoB**	-0.01(-0.07, 0.03)	0.47
**ApoE**	-0.12 (-0.93, 0.68)	0.74
**HOMA-IR**	-0.25 (-1.08, 0.57)	0.52
**HOMA-β**	-0.00 (-0.00, 0.00)	0.65
**CK**	-0.00 (-0.02, 0.01)	0.34

[1] Data from N=19 participants.
